# Zinc vs. Quinine

**Published:** 1879-08-20

**Authors:** W. Gibbon Carter

**Affiliations:** Richmond, Va.


### *ZINC vs. QUININE.*

BY W. GIBBON CARTER, M.D., RICHMOND, VA.

Owing to the high price of the alkaloids of cinchona, the disagreeable results, in some cases, from their administration, and their nauseous taste, I was induced, from the high praise given the sulphate of zinc in the treatment of intermitting fever by Prof. John Eberle, in his "Practice of Medicine," to give it a trial. Dr. Eberle says: "The sulph. zinc is an excellent remedy in the cure of intermittents. I have very rarely failed to arrest the disease as promptly with it as with quinine."

Dr. Frith, in a letter to Dr. S. Mitchell, of New York, dated Calcutta, 1805, speaks in the most favorable terms of this article as a remedy in intermittents. He asserts that while prescribing in the Philadelphia Dispensary, he found it to cure cases in which both the bark and arsenic had failed. Mr. Brand also observes that "in the cure of intermittents the sulphate of zinc is an admirable tonic."

I have, in a practice of over thirty years, had ample opportunity of giving the zinc a full and fair trial, and the gentlemen quoted above, I am satisfied, have not overestimated its curative power in this fever. Had I to decide which of the two, quinine or zinc, I would select to cure ague and fever, I would unhesitatingly select the latter. I have frequently promptly cut short the disease with the zinc after the quinine, freely used, had no more effect than pulverized brick dust. That its effects are far more lasting, and relapses seldom, I have been struck with.

While medical attendant to the Westham iron works, located five miles above Richmond, and on James river, and where paludal fevers were common, nine-tenths of the cases of intermittents were treated by zinc. So little quinine did I use with the operatives that I remember General Stone, who had a general supervision, was struck with it when looking over the bills for medicines ordered by me, and remarked, "I see, Doctor, where, before you had charge of the medical affairs here, we paid each year for eight, ten or twelve bottles of quinine, we pay now for one or two. How is this?" "I use sulphate of zinc as a substitute."

My mode of administering was simple. I have sometimes cut short —or rather, as the premonitory symptoms were coming on, thwarted— the paroxysm by giving the zinc in its emetic dose. Of course any other emetic would have done the same by its reaction. In the intermissions, I have ordered from two to three grains at a dose every three or four hours for adults, and to be continued some four days after the attacks have ceased. I generally give it in a little cold water. The addition of any of the flavored syrups, as lemon or ginger, would make a more agreeable dose.—*Southern Clinic.*

